# Myristicin and Elemicin: Potentially Toxic Alkenylbenzenes in Food

**DOI:** 10.3390/foods11131988

**Published:** 2022-07-05

**Authors:** Mario E. Götz, Benjamin Sachse, Bernd Schäfer, Andreas Eisenreich

**Affiliations:** Department of Food Safety, German Federal Institute for Risk Assessment (BfR), Max-Dohrn-Str. 8-10, 10589 Berlin, Germany; mario.goetz@bfr.bund.de (M.E.G.); benjamin.sachse@bfr.bund.de (B.S.); bernd.schaefer@bfr.bund.de (B.S.)

**Keywords:** alkenylbenzenes, myristicin, elemicin, safrole, methyleugenol, flavoring

## Abstract

Alkenylbenzenes represent a group of naturally occurring substances that are synthesized as secondary metabolites in various plants, including nutmeg and basil. Many of the alkenylbenzene-containing plants are common spice plants and preparations thereof are used for flavoring purposes. However, many alkenylbenzenes are known toxicants. For example, safrole and methyleugenol were classified as genotoxic carcinogens based on extensive toxicological evidence. In contrast, reliable toxicological data, in particular regarding genotoxicity, carcinogenicity, and reproductive toxicity is missing for several other structurally closely related alkenylbenzenes, such as myristicin and elemicin. Moreover, existing data on the occurrence of these substances in various foods suffer from several limitations. Together, the existing data gaps regarding exposure and toxicity cause difficulty in evaluating health risks for humans. This review gives an overview on available occurrence data of myristicin, elemicin, and other selected alkenylbenzenes in certain foods. Moreover, the current knowledge on the toxicity of myristicin and elemicin in comparison to their structurally related and well-characterized derivatives safrole and methyleugenol, especially with respect to their genotoxic and carcinogenic potential, is discussed. Finally, this article focuses on existing data gaps regarding exposure and toxicity currently impeding the evaluation of adverse health effects potentially caused by myristicin and elemicin.

## 1. Introduction

**Figure 1:**
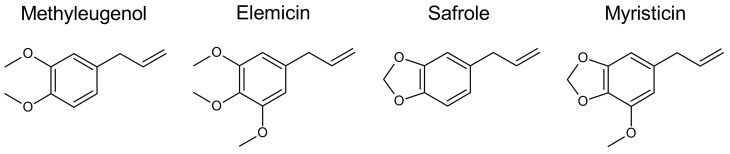
Structural formulas of methyleugenol, elemicin, safrole, and myristicin.

Myristicin (CAS N°: 607-91-0; IUPAC name: 4-methoxy-6-prop-2-enyl-1,3-benzodioxole), elemicin (CAS N°: 487-11-6; IUPAC name: 1,2,3-trimethoxy-5-prop-2-enylbenzene), safrole (CAS N°: 94-59-7; IUPAC name: 5-prop-2-enyl-1,3-benzodioxole), and methyleugenol (CAS N°: 93-15-2; IUPAC name: 1,2-dimethoxy-4-prop-2-enylbenzene) are secondary plant metabolites belonging to the group of alkenylbenzenes (Figure 1). Such derivatives may particularly be found in Umbelliferae (anise, star anise, fennel, sweet fennel, and parsley), Myristicaceae (nutmeg and mace), Labiatae (sweet and exotic basil), and Compositae (tarragon).

Several alkenylbenzene derivatives are known toxicants. For example, safrole and methyleugenol were classified by the International Agency for Research on Cancer (IARC) as “possibly carcinogenic to humans” (Group 2B) [1,2]. Moreover, the Scientific Committee on Food (SCF) of the European Commission (EC) considered safrole and methyleugenol as genotoxic carcinogens and suggested restrictions for their use in foods [3,4]. In consequence, the EC prohibited the addition of safrole and methyleugenol as pure flavoring substances to food and established maximum levels for these substances–when naturally present–for certain flavored foodstuffs, such as soups and sauces or non-alcoholic beverages (Regulation (EC) No 1334/2008). In contrast to safrole and methyleugenol, the structurally closely related alkenylbenzenes myristicin and elemicin were not assessed by international expert bodies in a comparable manner until now (Figure 1). This is probably due to the lack of reliable data regarding genotoxicity, carcinogenicity, and other toxic effects.

It is assumed that the toxicity of safrole and methyleugenol is mainly caused by metabolic activation at the allylic side chain, namely 1′-hydroxylation and subsequent sulfonation to the resulting allylic sulfate esters. These intermediates are instable and may subsequently react with cellular nucleophiles including DNA and proteins [5]. The relevance of further metabolites, e.g., quinone methides or other so far unknown reactive intermediates possibly involved in hepatotoxic effects are far less clear.

Paying attention to the chemical structure, it may be noted that the less intensively studied derivatives, myristicin and elemicin, each bear just one additional methoxy substituent at the benzene core as compared to safrole and methyleugenol, respectively (Figure 1). The allylic side chain is a common feature of all the four alkenylbenzenes discussed here. Metabolic differences with respect to the allylic side chain, the suspected site of metabolic activation leading to toxicity, are expected to be marginal. However, oxidative demethylenation of myristicin and oxidative demethylation of elemicin at the benzene core are not entirely comparable to similar reactions on safrole and methyleugenol. Consequently, the resulting patterns of urinary benzyl metabolites that could be detected in rats [6,7] and in humans [8] following elemicin and nutmeg ingestion, respectively, are different from those formed from safrole and methyleugenol in rodents.

Nevertheless, for all the four compounds, the toxic principle that is assumed to lead to toxification is the 1′-hydroxylation and subsequent sulfonation to the resulting allylic sulfate esters [5].

Intake of myristicin, elemicin, safrole, and methyleugenol is presumed to occur mainly from the consumption of spices and essential oils made thereof [9,10]. High levels may also be present in plant-based food supplements (PFS) as well as in various processed flavored foods such as sauces, baked goods, and beverages, such as cola-flavored softdrinks [5,10,11,12,13].

Taken together, the toxicological relevance of alkenylbenzene occurrence, especially for less intensively investigated members such as myristicin and elemicin, is still under discussion.

In this review, we summarize and discuss the current knowledge regarding occurrence, toxicokinetics, and the toxicity of myristicin vs. safrole, as well as elemicin vs. methyleugenol. Moreover, we highlight data gaps currently impeding the assessment of the adverse health effects of these substances.

## 2. Occurrence of Myristicin and Elemicin

The occurrence of the alkenylbenzenes safrole and methyleugenol in plants used as foods was described in detail elsewhere [5]. Beside this, aromatic plants, as well as powders, extracts, or essential oils made thereof can also serve as sources for myristicin and elemicin, which are described here.

Of note, when interpreting occurrence data, it should always be kept in mind that the chemical constituents of culinary spices, including alkenylbenzenes, are widely dependent not only on species but also on environmental factors such as geographic location, seasonal variation, and harvest time. Furthermore, the analytical method–especially the extraction procedure–may also affect the obtained occurrence data [14,15,16]. In the following part, as well as in Table 1, we will present relevant examples of (culinary) plants and their essential oils containing myristicin and elemicin.

### 2.1. Myristicin and Elemicin in Nutmeg and Mace

The name “myristicin” originally referred to the solids that crystallize from nutmeg oil while in prolonged storage. These, however, are today known to be myristic acid [17]. Elemicin was first identified as a component of the myristicin fraction from nutmeg oil [18].

The nutmeg tree is a tropical tree indigenous to the Maluku Islands of Indonesia (Myristica fragrans Houtt., family Myristicaceae). Its seeds consist of a kernel and a covering aril surrounding the kernel. Whereas mace designates the red lacy aril, the dried kernels of the ripe seeds are named nutmeg. When grounded material or powders are hydrodistilled, about 2.4% crude oil can be obtained [19]. These oils are rich in alkenylbenzenes, such as eugenol (19.9%), methyleugenol (16.7%), methyl iso-eugenol (16.8%), myristicin (2.3%), safrole (1.6%), and elemicin (1.7%). In contrast, a slightly different composition of oils from the dried kernels of Myristica fragrans originating from Sri Lanka was reported, with almost no eugenol (0.2%), or methyleugenol (0.6%), nearly equal amounts of safrole (1.4%) and elemicin (2.1%), but higher levels of myristicin (4.9%) [20]. Numerous reports on the composition of nutmeg oils are published, reporting varying levels of alkenylbenzenes in nutmeg seeds. It is, on the one hand, the storage of ground powders [21], but on the other hand, very much the geographical origin that determines the volatile composition of nutmeg extracts as recognized by Baldry and colleagues [22]. They showed high variabilities in alkenylbenzene contents, with myristicin ranging from 0.5% to 12.4%, safrole from 0.1% to 3.2%, elemicin from 0.3% to 4.6%, methyleugenol from 0.1% to 1.2%, and eugenol from 0.1% to 0.7% in different nutmeg oils from West India and South East Asia. Mace powders and mace oils contain similar constituents as nutmeg powders and nutmeg oils. For example, in 10 powdered genuine Indonesian nutmeg seeds extracted with boiling methanol, myristicin accounted for up to 2.9% and safrole accounted for up to 0.39%. Nutmeg oil from Indonesian nutmegs contained 9.73% myristicin and 2.16% safrole [23].

Nutmeg and mace are used as domestic spices and as flavoring ingredients in many food products, such as in gelatins, puddings, sweet sauces, baked goods, meats, fish, pickles (processed vegetables), candy, ice cream, and non-alcoholic beverages [24,25]. In addition, several globally available PFS contain nutmeg seed powders or nutmeg oils to very varying extents [11].

Further relevant examples for plants and their essential oils containing myristicin and elemicin, as well as other selected alkenylbenzenes, are listed in Table 1.

**Table 1:** Occurrence of safrole, myristicin, methyleugenol, and elemicin found in essential oils (EO) from culinary plants.

Source	Safrole	Myristicin	Methyleugenol	Elemicin
Nutmeg	0.1–3.2% [19,20,22,23]	0.5–12.4% [19,20,22,23] (16.9 ± 0.6 mg [26])	0.1–16.7% [19,20,22]	0.3–4.6% [19,20,22]
Parsley		20.3–94.1% (seed) [27,28]; 3.1–91.9% (leaf) [27]; 6.6–30.1% (root) [27] (1435 ppm [29]; 3.6–526 ppm (leaf) [30])		
Sweet fennel		2.5–10% (root) [31,32]		
Dill		0.21% (seed) [33]; 4.38% (root) [32]		
Parsnip		18.3–66.2% (root) [34,35] (200 ppm (root) [36])		
Sweet basil			9.24–87.04% [15]; 0.03% (flower) [37,38]; 0.06% (stem) [37,38]; 0.18–76% (leaf) [37,38,39]	0.30% (stem) [37,38]
Carrot		34.4% (leaf) [40]; 43.9% (fruit) [40]; 0.4–29.7% (root) [41,42]; (0.5–15 ppm (root) [43])	2.51% (fruit) [44]	1.4–35.3% [45]; 32.89% (fruit) [44]
Pepper (Piper)	0.2–3.0 mg/kg (fruit) [46]; <3% (fruit) [47]; 4.81% (fruit) [48]; 24% [49]; 49% [50]; 64–98% [51]	<1% (fruit) [47]; 16.55% (fruit) [48]; 0.3–7.6% (leaf) [52]	<3% (fruit) [47]; 1.53% (fruit) [48]	<1% (fruit) [47]; 3.91% (fruit) [48]; 0.2–1.6% (leaf) [52]
Japanese star anise	6.6% [53]	3.5% [53]	9.8% [53]	
Tarragon			9.59–28.40% (seeds) [54]	21.45–38.90% (seeds) [54]
Sweet bay			3.1% (flower) [55]; 4.7% (bark) [55]; 16.0% (stem) [55]; 11.8–21.3% (leaf) [55,56]	0.8% (stem) [55]; 5% (leaf) [56]

### 2.2. Myristicin and Elemicin in Food Flavorings

Due to the intentional use of essential oils and the dried powder of nutmeg or mace for flavoring reasons, certain types of soft drinks, pastries, and some types of crisps contain high levels of myristicin and elemicin.

Cola-flavored soft drinks may contain nutmeg oil and/or mace oil, which consist of different major compounds, such as sabinenes and myrcene, as well as at least five different alkenylbenzenes. Myristicin, safrole, and elemicin mainly determine the flavor of these oils. Accordingly, myristicin, safrole, elemicin, methyleugenol, and eugenol were detected in cola-flavored soft drinks [57]. In 2013, Raffo et al. published quantitative data on the amounts of safrole and myristicin in the cola-flavored soft drinks of different brands following different processing procedures, including various storage conditions. Levels of safrole and myristicin varied approximately 2–3 orders of magnitude. In flavored soft drinks, average concentrations of safrole and myristicin were 23.0 and 168.3 µg/L, with minimum contents of 0.6 and 0.4 µg/L and maximum levels of 43.9 and 325.6 µg/L, respectively [12]. These variations might be due to variable levels of alkenylbenzenes in the added essential oils. For example, measurements of alkenylbenzene concentrations in different nutmeg oils of specific geographical origins revealed an at least 30-fold variation, e.g., in the levels of safrole (ranging from 0.1 to 3.2%) and myristicin (0.5 to 13.5%), respectively [57]. In the study of Raffo et al., only the levels of myristicin and safrole were measured in cola-flavored soft drinks, but not those of other alkenylbenzenes. Therefore, the total amount of alkenylbenzenes in cola-flavored soft drinks remains unknown so far.

Another important example for processed foods containing alkenylbenzenes is “*Pesto*”. This traditional dish from Genova, Italy, mainly consists of olive oil, hard cheese, pine nuts, garlic, salt, and basil leaves. Different alkenylbenzenes were detected at varying levels in basil-containing “*Pesto*”, including methyleugenol (22.9–56.4 mg/kg), myristicin (13.2–15.8 mg/kg), estragole (3.2–34.1 mg/kg), and apiol (3.4 mg/kg) [58].

Parsley and dill teas can be purchased without restriction. Recently levels of alkenylbenzenes in such teas were investigated. Myristicin, methyleugenol, apiol, and estragole are detected to varying extents in dry tea samples or in hot water herbal extracts containing parsley, dill leaves, or seeds, or being in a mixture with other herbs. The total amount of alkenylbenzenes in the dry tea samples ranged from 18 to 1269 μg/g dry preparation [59]. In 2017, Alajlouni and colleagues also found relevant levels of the alkenylbenzenes myristicin, apiol, and estragole (17–6487 μg/g) in parsley and dill-based PFS [60].

Beside this, baked goods, meat products, condiments, relishes, soft candy, gelatin, pudding, soups, alcoholic beverages, and gravies may also contain myristicin and elemicin to various and often unknown amounts if refined with oils from parsley, nutmeg or mace [24,61]. This also applies to other alkenylbenzenes [62,63]. Therefore, monitoring of myristicin, elemicin, and other alkenylbenzenes in many food commodities appears justified in order to gain a reliable database for future exposure assessments [25].

### 2.3. Myristicin and Elemicin in Foods

Analytical methods are already in place to monitor myristicin and elemicin in complex food matrices [25]. An early study reported 16.9 mg myristicin per gram dried nutmeg powder following 12 h methanol extraction at 50 °C [26]. Other methods for analyzing ground nutmeg, wine and beer spices, and many food commodities utilize ultrasonic assisted extractions, followed by solid phase extraction and gas chromatography (GC)–mass spectrometry (MS) [64,65]. Other methods for analyzing myristicin from ground nutmeg (502 µg/g), from wine and beer spices (11.87 µg/g), from some food commodities (2.46–15.22 µg/g), and even from human serum (17.60–33.25 µg/g from human volunteers who incorporated 100 mg myristicin 1 h before blood sampling) utilize ultrasonic assisted extractions, followed by solid phase extraction and gas chromatography (GC)–mass spectrometry (MS) [64,65]. Other methods use functionalized magnetic microspheres for isolation of allyl-benzodioxoles, followed by gas chromatography–mass spectrometry, such as myristicin (264.2–599.6 µg/L) and safrole (14.0–40.35 µg/L) from cola drinks [66]. However, in these methods, varying and often not fully validated analytical procedures were used, hampering the comparability of the analytical results. In addition, for many food categories no data are available at all. Since there is no legal mandate for monitoring all potentially toxic alkenylbenzenes in all relevant food categories, the availability of comprehensive and reliable occurrence data is currently rather limited. Taken together, the actual occurrence levels of myristicin and elemicin, as well as of many other alkenylbenzenes, are still widely unknown for many foods.

## 3. Toxicity of Myristicin and Elemicin: Lessons Learned from Safrole and Methyleugenol

In the following, the current knowledge on the toxicity of myristicin and elemicin in comparison to the structurally related and well-investigated alkenylbenzenes, safrole and methyleugenol, is summarized.

### 3.1. Metabolism of Myristicin and Elemicin vs. Safrole and Methyleugenol

#### 3.1.1. Common Structural Features

Initial steps of the hepatic activation of methylenedioxy- and methoxy-substituted allylic alkenylbenzenes include epoxidation of the exocyclic double bond followed by its cleavage by microsomal or cytosolic epoxide hydrolases or spontaneous hydration to generate 2′,3′-dihydrodiols [67]. Such metabolites are detected in the urine of animals treated with allylbenzenes [6,68,69,70]. Another pathway may be the hydroxylation of the 1′-carbon atom adjacent to this 2′,3′-double bond [71]. Side chain reactions of alkenylbenzenes are catalyzed by various cytochrome P450 monooxygenases (CYPs). Epoxides and dihydrodiols may be derived not only from the allylbenzene compounds but also from some of their metabolites, which still possess an intact allyl group, such as the allylcatechols [72]. However, phenolic and catecholic compounds typically undergo rapid phase II conjugation, which might be a predominant pathway for such metabolites as also shown for the alkenylbenzene eugenol containing a free phenolic group [73]. Thus, in contrast to alkenylbenzenes that bear only methoxy or methylenedioxy substituents, the high first-pass conjugation and rapid elimination may explain why eugenol is deemed to be less toxic as compared to the well-known hepatocarcinogens methyleugenol and safrole.

**Figure 2:**
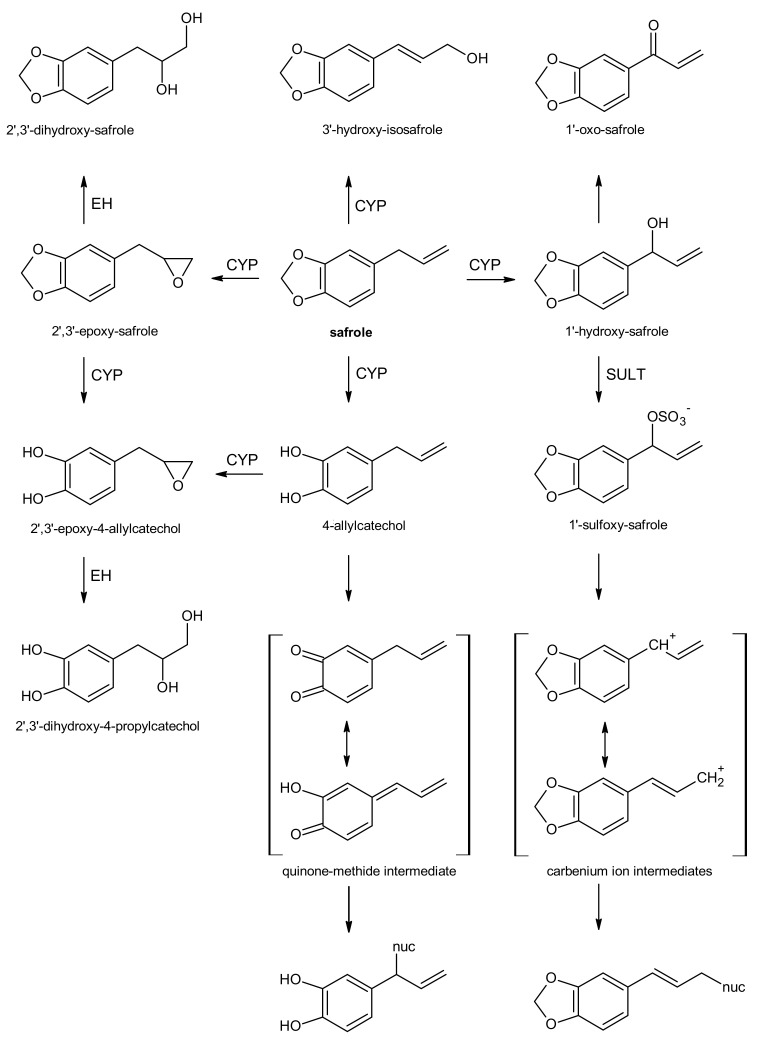
Metabolite excretion of safrole in the rat is reported to be 93% within 72 h, and most of this material (86%; [79]) would consist of metabolites formed via demethylenation of the methylenedioxy moiety to yield carbon monoxide or formate and the dihydroxy-benzene moiety [80]. The other metabolic routes observed were allylic hydroxylation and the epoxide-diol pathway [70,79]. Oxidations of the allylic side chain of safrole may proceed (i) via an epoxide resulting in side chain propane diols during different stages of the metabolic steps [72], or (ii) via 1′-hydroxylation followed by sulfonation that might lead to a reactive carbocation intermediate [5]. Other possible steps of metabolic ways of safrole are (iii) the subsequent oxidation of the 1′-hydroxysafrole to the 1′-oxo-safrole [81], (iv) oxidation at the 3′-position to yield 3′-hydroxy-isosafrole, and (v) the demethylenation of safrole to 4-allylcatechol that may isomerize to its quinone-methide [82,83,84]. The occurrence of glutathione conjugates at the 1′-position may be indicative of the intermediate formation of *para*-quinone methide tautomers [82], whereas glutathione conjugates at the benzene ring point to reactions with *ortho*-quinone intermediates [82]. CYP: cytochrome P450 monooxygenases; SULT: sulfotransferases; EH: epoxide hydrolases; nuc: nucleophilic structures such as DNA or proteins.

Following hydroxylation at the 1′-position (Figure 2), the alcoholic metabolite can be sulfonated. Subsequent heterolytic cleavage of the formed sulfate moiety would generate an electrophilic carbenium ion intermediate, which is highly reactive towards nucleophilic sites [74,75], and that may, for example, generate glutathione (GSH) conjugates, as well as adducts with proteins, RNA, or DNA [76]. Since the carbenium ionic charge is delocalized, adducts can be formed at the 1′- or 3′-position, with the 3′-position being the preferred site [77,78].

However, the metabolic pathway to the carbenium ions is only one selected pathway, already often discussed with respect to the cyto- and genotoxic activity of alkenylbenzenes. This metabolic pathway presumes the presence of sulfotransferases (SULT) and cofactors such as 3′-phosphoadenosine-5′-phosphosulfate (PAPS) [5].

On the other hand, alkenylbenzenes and their metabolites that bear ortho- and/or para-phenolic groups may form quinone methide intermediates (Figure 2) [82,85] that are also prone to be conjugated by GSH or react directly with other nucleophiles in the cell. The transient formation of a quinone methide of eugenol appears plausible [85] since an eugenol GSH conjugate was detected utilizing rat liver or rat lung microsomes [86]. The cytotoxic effects of eugenol recognized in rat hepatocytes are reasoned to be due to the formation of a reactive quinone methide intermediate [87]. In 1990, Fischer et al. tentatively identified metabolites including thiophenol metabolites (11%) following eugenol ingestion in the urine of humans, presumably formed by GSH conjugation at an aromatic ring position [73]. Thus, methoxylated non-phenolic substances (e.g., methyleugenol and elemicin) may as well undergo CYP enzyme-mediated *O*-demethylation and subsequent quinone methide formation followed by GSH conjugation. Similarly, there can be oxidative demethylation of methoxy groups in elemicin by CYP1B1 [88], creating the possibility to yield also catechols or other phenols and conjugates, as was shown from benzodioxole-substituted alkenylbenzenes myristicin and safrole in rat and human urine using GC-MS [8].

A number of CYP isoenzymes are capable of catalyzing the 1′-hydroxylation of akenylbenzenes [88,89,90,91,92,93]. An overview of CYPs that are demonstrated to be involved in the oxidation of methyleugenol, elemicin, safrole, and myristicin is shown in Table 2.

Apart from the epoxide, the carbenium ion, and the quinone methide metabolic pathways of the alkenylbenzenes already discussed, another metabolic pathway that may occur after rearrangement of the double bond from 2′,3′-position to 1′,2′-position is the oxidation of 3′-hydroxy metabolites of alkenylbenzenes leading to cinnamic acids and propionic acids [6,68,69]. In principle, 3′-hydroxy-1′,2′-propenylbenzenes may be equivalent to 1′-hydroxy-allylbenzenes as substrates for hepatic SULTs. On the other hand, due to steric reasons further side chain oxidation of the 3′-hydroxy-propenylbenzenes yielding cinnamaldehydes and cinnamic acids, which can be conjugated with GSH or glycine, appear to dominate. Further oxidation, probably via the fatty acid *β*-oxidation cycle, would lead to side chain cleavage and the formation of benzoic acids and its glycine conjugates [5].

Seemingly small, but relevant structural molecular differences in benzene ring substituents of the parent alkenylbenzenes call for a closer look at the potential metabolic pathways of elemicin, myristicin, methyleugenol, and safrole. In an attempt to identify similarities and possible differences of elemicin and myristicin, we compared their metabolic features to the closely related derivatives methyleugenol and safrole. Those two compounds bearing only methoxy groups at the benzene ring without methylene bridge are methyleugenol and elemicin. The two compounds with a methylenedioxy moiety are safrole and myristicin, which are categorized as benzodioxoles.

#### 3.1.2. Metabolism of Methyleugenol

Results of ADME experiments performed in 2000 within a study of the National Toxicology Program (NTP) led to the conclusion that absorption of orally ingested methyleugenol in rats and mice is rapid and complete, and that the distribution of methyleugenol to tissues is fast. In rodents, methyleugenol is extensively metabolized in the liver and more than 70% of the dose administered is found in the urine of rats and mice as hydroxylated, sulfated, or glucuronidated metabolites [92].

With view on the toxicity of methyleugenol, it is generally assumed that bioactivation is mainly mediated via 1′-hydroxylation at the allylic side chain followed by sulfo conjugation, yielding a highly reactive sulfate ester [10,94].

In the NTP study, it was shown that repeated ingestion of methyleugenol may saturate metabolic enzymes [92], leading to greater tissue accumulation and thus higher probability for genotoxicity, mutations, and malignant cell transformations. Saturability of metabolism is of special concern in cases when 1′-hydoxylation of the allylic side chain becomes more prominent over other pathways. This may enhance hepatocarcinogenesis in rodents at higher dose levels [95].

In rat bile, methyleugenol could be found in the form of GSH conjugates. These conjugates detected by Yao and colleagues potentially resulted from reactions with methyleugenol-derived epoxide metabolites, α,β-unsaturated aldehydes, carbenium ions, and quinone methides [96]. These conjugates were further metabolized, yielding the cysteine conjugates found in rat urine. In GSH-fortified microsomal preparations that lack SULT and PAPS, it was generally not expected that carbenium intermediates would be formed. However, Yao et al. found 1′-bound GSH and related cysteine conjugates in such incubations [96]. Thus, it is hypothesized that 1′-hydroxy metabolites or other metabolites than the sulfate esters may directly react with GSH under certain conditions.

Beside 1′-hydroxylation, the metabolites observed in rats and mice suggest that methyleugenol can also undergo demethylation, ring, and/or further side chain oxidations [92].

The NTP authors further concluded that the risk to humans ingesting methyleugenol is expected to be subject to marked inter-individual metabolic variability. Indeed, hydroxylation of methyleugenol investigated in human liver microsomes varied considerably (37-fold), with the highest hydroxylation rate being similar to that observed with liver microsomes from rats [97]. Moreover, one study by Tremmel et al. demonstrated that methyleugenol-induced DNA adduct levels in human liver samples were dependent on the SULT1A1 copy number [94].

#### 3.1.3. Metabolism of Elemicin

Elemicin is the natural continuation of methyleugenol, bearing two *meta*- and one *para*-methoxy group relative to the allyl side chain. For this compound, the *O*-demethylation pathway becomes more prominent, which leads to some divergent metabolites, compared to methyleugenol. In 1980, Solheim and Scheline revealed that the two major metabolic pathways of elemicin in rats follow the cinnamoyl pathway and the epoxide-diol pathway [6]. The former route gives 3,4,5-trimethoxyphenyl-propionic acid and its glycine conjugate as major urinary metabolites, whereas 2′,3′-dihydroxy-elemicin is the most prominent metabolite of the latter route. In addition, elemicin can also be 1′-hydroxylated at the allylic side chain. When comparing the kinetic constants for conversion of elemicin and 1′-hydroxy-elemicin by male rat liver and mixed gender pooled human liver fractions, van den Berg et al. concluded that glucuronidation of 1′-hydroxy-elemicin, representing a detoxification pathway, is the most important pathway in rats and in humans. In contrast, bioactivation of 1′-hydroxy-elemicin by sulfonation was suggested to be only a minor pathway in both rat and human liver [76].

In 2019, Wang et al. confirmed and extended these studies. They found a total of 22 metabolites for elemicin in mice, e.g., in urine, feces, and plasma [88]. In vivo, elemicin and most of its metabolites were mainly excreted in urine collected from 0 to 24 h post-procedure in metabolic cages of male C57BL/6 mice that were orally administered 100 mg/kg elemicin. The obtained results indicate that phase I metabolic reactions of elemicin included demethylation, hydroxylation, hydration, allyl rearrangement, reduction, hydroformylation, and carboxylation. Phase II metabolism of elemicin yielded several conjugates, e.g., with cysteine, *N*-acetyl cysteine, glucuronic acid, glycine, or taurine [88]. In addition, the 4-demethoxylated forms of elemicin and of 2′,3′-dihydroxy-elemicin could be detected in human urine after nutmeg abuse [8].

#### 3.1.4. Metabolism of Safrole

From a toxicological point of view, safrole bioactivation by sequential 1′-hydroxylation and sulfonation, resulting in reactive sulfate esters capable of forming adducts with cellular nucleophiles such as DNA, is of high relevance [71,89]. In 1983, Boberg et al. identified 1′-sulfoxy-safrole as an ultimate electrophilic metabolite of safrole and as an initiator of hepatic carcinogenicity in vivo. The toxicological relevance of this pathway was demonstrated in mice co-treated with the hepatic SULT inhibitor pentachlorophenol (0.05% added to the diet of mice) in vivo and in mice being genetically defective with respect to the hepatic synthesis of PAPS [75].

However, work in rats, mice, and guinea pigs elucidated multiple metabolic pathways of safrole far beyond 1′-hydroxylation and sulfo conjugation. Upon intraperitoneal (i.p.) injection safrole is metabolized in rat and guinea pig by the epoxide-diol pathway and by cleavage of the methylenedioxy ring to form a catechol [98,99]. Since an allylic double bond is still present in the catechol and 1′-hydroxy-safrole, both metabolites can be further metabolized via epoxides to the corresponding dihydrodiols. A small amount of a triol 1′,2′,3′-trihydroxy-safrole was found in rat urine by Stillwell and colleagues [70]. Interestingly, 2′,3′-epoxy-safrole apparently has sufficient stability in vivo to be absorbed from the peritoneal cavity to the circulatory system, and to persist even in urine. The major urinary metabolites identified by GC-MS were 4-allylcatechol, 1′-hydroxy-safrole, 2′,3′-dihydroxy-safrole, 2′,3′-dihydroxy-4-propyl-catechol, 2′-hydroxy-1′- (3,4-methylenedioxy-phenyl)-propanoic acid, and 3,4-methylenedioxy-benzoyl glycine [70] (Figure 2).

Urinary metabolites of safrole in the rat were also identified via GC-MS in a further study performed in 1982. Metabolite excretion was 93% within 72 h, and most of this material (86%) consisted of metabolites formed via demethylenation of the methylenedioxy moiety. The other metabolic routes observed were allylic hydroxylation and the epoxide-diol pathway [79].

#### 3.1.5. Metabolism of Myristicin

Myristicin is well absorbed following oral exposure and is metabolized extensively.

Metabolism of the volatile alkenylbenzene myristicin results in the formation of less volatile metabolites, predominantly remaining in the aqueous phase on extraction with ether [99].

Early experiments highlighted the cleavage of the methylenedioxyphenyl moiety concomitant with CO_2_ release from myristicin as an important metabolic pathway. Within 48 h after oral administration of radiolabeled myristicin to male albino mice, 73% of the radiocarbon was set free as ^14^CO_2_ [98], which was potentially formed from the hydroxylation of the methylene group of myristicin and subsequent release and degradation of formate-^14^C. This demethylenation reaction was found to be catalyzed by microsomal CYPs and would yield the corresponding catechol derivative.

Later analytical studies in rat and human urine indeed revealed further water-soluble metabolites of myristicin, including the catecholic derivatives. In male Wistar rats that were administered myristicin once by oral gavage (100 mg/kg), different metabolites were identified in urine, including 1′-hydroxy-myristicin, 5-allyl-2,3-dihydroxy-1-methoxy-benzene, 5-allyl-2-hydroxy-1,3-methoxy-benzene, 5-allyl-1-hydroxy-2,3-methylendioxy-benzene, 5-(2′,3′-dihydroxypropyl)-1-hydroxy-2,3-methylendioxy-benzene [8].

Incubation of myristicin in rat liver microsomes formed two major metabolites, 1′-hydroxy-myristicin and 5-allyl-1-methoxy-2,3-dihydroxy-benzene, bearing a catechol moiety [7]. Those metabolites were also identified in the above mentioned study by Beyer et al. in 2006 [8].

Isolation of metabolites from male Sprague–Dawley rat urine after a single oral administration of 100 mg/kg myristicin, and comparison before and after glucuronidase treatment, suggests that the catecholic metabolites 5-allyl-1-methoxy-2,3-dihydroxy-benzene and 1′-hydroxy-myristicin are also excreted in their respective conjugated forms [7].

Currently, no comprehensive studies with respect to quantitative metabolism and excretion of myristicin in humans are available. However, one study examined metabolites present in the urine of a patient who ingested five nutmeg seeds, resulting in an intoxication [8].

### 3.2. Genotoxicity

As described above, different metabolic pathways may lead to the formation of reactive intermediates capable of binding DNA, thereby causing genotoxicity. For many alkenylbenzenes, it is widely accepted that the 1′-hydroxylation at the allylic side chain, followed by SULT-mediated sulfo conjugation yielding a highly electrophilic sulfate ester, might be the most relevant pathway leading to toxicity [10]. The sulfate ester may form inter alia DNA adducts as demonstrated by ^32^P-postlabeling techniques and mass spectrometry [78,100,101,102,103,104,105]. Structures of four DNA adducts formed in mouse liver after administration of the proximate hepatocarcinogen 1′-hydroxy-estragole were initially described by Phillips et al. in 1981 [106,107]. Similar kinds of studies, as well as studies on other genotoxicity endpoints and mutagenicity, were performed for many alkenylbenzenes, as systematically reviewed in detail elsewhere [5,10,108]. In the following part, the most relevant studies on genotoxicity of methyleugenol, elemicin, safrole, and myristicin are exemplarily described in brief.

#### 3.2.1. Genotoxicity of Methyleugenol vs. Elemicin

Methyleugenol was found to induce sister chromatid exchange (SCE) in Chinese hamster ovary (CHO) cells after metabolic activation, as well as intrachromosomal recombination in yeast with and without metabolic activation [92]. Some years later, Groh and colleagues further characterized the impact of methyleugenol and its metabolites on DNA damage induction in vitro. It was observed that 1′-hydroxy-methyleugenol and 2′,3′-epoxy-methyleugenol had a higher DNA strand breaking activity than the parent compound methyleugenol in Chinese hamster lung fibroblast (V79) cells, demonstrating the marked relevance of these metabolites. However, in the same study, only 3′-oxomethylisoeugenol and 2′,3′-epoxy-methyleugenol induced the formation of micronucleated V79 cells [109]. Furthermore, methyleugenol and the oxidative metabolites concentration dependently increased the amount of DNA strand breaks, as measured using the in vitro alkaline comet assay in human colon carcinoma HT29 cells [110,111].

In 1992, Chan and Caldwell found that methyleugenol, 1′-hydroxy-methyleugenol and 2′,3′-epoxy-methyleugenol caused unscheduled DNA synthesis (UDS) in rat hepatocytes, and that the inducing potency of the 1′-hydroxy metabolite was higher than that of the parent substance in vitro [112]. In 2006, methyleugenol was also shown to form DNA adducts after hydroxylation and sulfonation. DNA adducts of methyleugenol were detected using ^32^P-postlabeling techniques in the livers of F344 rats (*n* = 4 out of 8) exposed orally to 5 mg/kg/day for 28 days. No adducts were found after exposure to 1 mg/kg/day [113].

In 2013, Herrmann et al. detected methyleugenol-induced DNA adducts also in human liver samples [114]. Twenty-nine human liver samples unambiguously contained the *N*^2^- (*trans*-methylisoeugenol-3′-yl)-2′-deoxyguanosine adduct (*N*^2^-MIE-dG). A second adduct, *N*^6^- (*trans*-methylisoeugenol-3′-yl)-2′-deoxyadenosine (*N*^6^-MIE-dA), was also found in most samples, but at much lower levels. The median methyleugenol DNA adduct level detected in human non-tumorous liver samples was 13/10^8^ nucleotides for *N*^2^-MIE-dG and *N*^6^-MIE-dA combined, corresponding to 1700 adducts per diploid genome (6.6 × 10^9^ base pairs). As further elegantly reported, hepatic DNA adduct formation by methyleugenol in mice is strongly affected by their SULT1A content [115,116], proving the toxicological relevance of this metabolic pathway. Indeed, also in human liver samples, an association between the SULT1A1 copy number and the adduct level was demonstrated [94]. Moreover, it is shown in vitro for the structural derivative estragole that the resulting DNA adducts are inefficiently repaired [117], which might contribute to the accumulation of substantial levels of DNA adducts upon prolonged dietary exposure.

Beside this, Yang et al. recently showed that reactive metabolites of methyleugenol were also able to form RNA adducts [118]. However, the biological consequences of these RNA adductions are so far unclear, as also mentioned by the authors.

As shown for methyleugenol [112], also elemicin was found positive in a DNA binding assay and in UDS assays [24,76,119,120].

Despite the well-recognized DNA damages, methyleugenol is reported to be only weakly or non-mutagenic in different bacterial test systems with or without metabolic activation [3,92,121,122]. In another study done by Groh et al. in 2012, it was shown that methyleugenol did not cause mutations at the *hprt* locus in cultured V79 cells after 1 h of incubation. After extended treatment (24 h) only 2′,3′-epoxy-methyleugenol exhibited slight mutagenic activity with a mutation frequency being 4–5 times higher than the spontaneous mutation frequency of the solvent control [109]. A possible explanation for the lack of mutagenicity, especially in bacterial systems, might be the lack of metabolic competence, especially in view on SULTs or the cofactor PAPS [5,122].

The mutagenic potential of methyleugenol was also studied in vivo [3]. Data published by NTP indicates that oral administration of methyleugenol via gavage (10–1000 mg/kg bw; 5 days/week) to B6C3F1 mice does not cause micronucleus formation in peripheral blood erythrocytes [92]. Likewise, it was unable to induce chromosomal aberrations in CHO cells or micronucleus formation in peripheral blood erythrocytes of mice in other studies [92,122]. In contrast, Devereux and colleagues observed a higher frequency of β-catenin gene mutations (20/29; 69%) in hepatocellular carcinomas of mice exposed to methyleugenol (37–150 mg/kg) than in spontaneous liver tumors (2/22; 9%) from unexposed mice [123]. Since deregulation of Wnt/β-catenin signaling is considered an early event in chemically induced hepatocarcinogenesis, this observation represents an indication of the genotoxic potential of methyleugenol [3,122].

Beside this, mutagenicity of methyleugenol was recently verified in vivo, utilizing a xanthine-guanine phosphoribosyltransferase (*gpt)* delta rodent gene mutation assay [124]. For this in vivo mutation assay, transgenic *gpt* delta rats (*n* = 10/group, both sexes) were treated for 13 weeks with different doses of methyleugenol via gavage (0, 10, 30, and 100 mg/kg). A significant increase in mutagenicity assessed, via *gpt* and *Spi^−^* mutant frequencies, was observed in rat hepatocytes of the highest dose group. Mutant frequencies were further associated with pro-carcinogenic processes. From these data, the authors concluded that genotoxic mechanisms might be involved in methyleugenol-induced hepatocarcinogenesis [124].

In contrast to methyleugenol, there is currently no literature available regarding the mutagenic potential of elemicin. However, the structural features and the few data on genotoxicity suggest such an activity also for elemicin.

#### 3.2.2. Genotoxicity of Safrole vs. Myristicin

The genotoxic activity of safrole is known. For example, it was demonstrated that safrole is capable of inducing sister chromatid exchanges, chromosomal aberrations, replicative DNA synthesis, and DNA adducts in rat liver in vivo [125]. It appears that these effects result from the 1′-hydroxylation followed by sulfo conjugation yielding reactive sulfate esters. This is because the concomitant application of the SULT-inhibitor PCP or the use of brachymorphic mice, being deficient in the SULT cofactor PAPS, strongly reduced the genotoxic effects [126].

Already in 1986, Reddy and Randerath reported that two DNA adducts were detected by ^32^P-postlabeling techniques in the liver of adult female CD1 mice treated with safrole [104]. These DNA adducts were identified as *N*^2^- (*trans*-isosafrol–3′-yl)-2′-deoxyguanosine and *N*^2^- (safrol-1′-yl)-2′-deoxyguanosine. In 1998, using the same ^32^P-postlabeling assay, Daimon et al. studied DNA adduct formation in hepatocytes isolated from male F344 rats exposed to safrole [127]. The sum of the two above mentioned major DNA adducts was 898 DNA adducts/10^8^ nucleotides. In this study, hepatocytes were isolated 24 h after a single dose of safrole or five repeated doses (once a day) by gavage and allowed to proliferate in Williams′ medium E supplemented with an epidermal growth factor. This enabled a certain percentage of DNA repair in situ. Beside this, safrole was shown to cause UDS in cultured rat hepatocytes, but not in HeLa cells [128,129].

Randerath et al. investigated the DNA adduct formation of a series of alkenylbenzenes in the liver of adult female CD-1 mice by ^32^P-postlabeIing 24 h after i.p. administration of non-radioactive test compounds (2 or 10 mg/mouse). The known hepatocarcinogens, safrole, estragole, and methyleugenol, exhibited the strongest binding to mouse liver DNA. However, the formation of DNA adducts in the liver were demonstrated also for myristicin in male B6C3F1 mice and female CD-1 mice. In comparison to safrole, estragole, and methyleugenol, substitution at the 3-, 4-, and 5-positions of the benzene ring of allylbenzenes (elemicin, myristicin) results in compounds with intermediate DNA binding capability [100]. In 2007, Zhou et al. further proved that myristicin forms DNA adducts comparable to those of safrole and methyleugenol in cultured human hepatocytes as well as in adult mouse liver, as analyzed via ^32^P-postlabeling [130]. With the exception of methyleugenol, DNA adduction was dose-dependent in these experiments, decreasing in the order, methyleugenol > safrole ~ myristicin.

In other experiments, female mice were exposed to soft drinks. Covalent liver DNA adducts detected by ^32^P-postlabeling were identical to those detectable with the single compounds myristicin and safrole. Liver adduct levels increased with exposure duration [101].

DNA adduct formation and DNA damage by myristicin were also assessed using an avian egg model [131,132,133]. Medium white turkey eggs with 22- to 24-day-old fetuses received three injections of nine alkenylbenzenes: safrole (1, 2 mg/egg), methyleugenol (2, 4 mg/egg), estragole (20, 40 mg/egg), myristicin (25, 50 mg/egg), elemicin (20, 50 mg/egg), anethole (5, 10 mg/egg), methyl isoeugenol (40, 80 mg/egg), eugenol (1, 2.5 mg/egg), and isoeugenol (1, 4 mg/egg). Fetal livers were harvested 3 h after the last injection. Measurements of DNA strand breaks were executed using the comet assay and DNA adduct formation, and were analyzed via ^32^P-postlabeling. At the highest doses tested, estragole, myristicin, elemicin, safrole, methyleugenol, and anethole induced DNA adduct formation. Estragole, myristicin, and elemicin also induced DNA strand breaks as measured with the comet assay.

In freshly isolated hepatocytes from male F344 rats, myristicin induced a dose-dependent but slight increase in UDS, an indicator of DNA excision repair activity [120]. However, the authors concluded from the obtained data that myristicin was negative in that assay [120]. Decreased DNA damage repair might be an important indirect genotoxic mode of action, as highlighted by Martins et al. in 2014 and 2018 [10,134]. They showed in vitro that exposure of human leukemia cells (K562) for 6 h with 100 µM myristicin led to reduced expression of various DNA damage response genes including *OGG1* (base excision repair), *ERCC1* (nucleotide excision repair), *RAD50* (double strand break repair), *ATM* (DNA damage signaling), and *GADD45G* (stress response). As summarized by Célia Maria da Silva Martins in 2016 in her dissertation, myristicin appears to activate apoptotic mechanisms and downregulate DNA damage response genes involved in nucleotide excision repair, double strand break repair, DNA damage signaling, and stress response [135]. In 2011, Martins et al. studied the mutagenic potential of myristicin in vitro in mammalian cells [136]. In this experimental setting, myristicin tested without metabolic activation was negative in a comet assay used to evaluate DNA breaks, as well as in a γH2AX assay (sometimes recognized as an indicator for DNA double strand breaks) performed in CHO cells.

The DNA damaging activity may lead to the manifestation of heritable mutations. The mutagenic potential of safrole and its metabolites was studied in different experimental settings [4]. In the bacterial reverse mutation assay (Ames test), safrole was generally negative, or at most, weakly positive [137,138,139]. In contrast to the parent compound safrole, 1′-hydroxy-safrole, as well as other metabolites (2′,3′-epoxy-safrole, 1′-acetoxysafrole and 1′-oxo-safrole), were demonstrated to be directly mutagenic in the Ames test [139,140]. In addition, safrole was shown to be mutagenic in other experimental settings (bacteria and yeast) and to induce cell transformation in vitro [141,142]. The mutagenic potential of safrole, including the induction of gene mutations, chromosomal aberrations, DNA single-strand breaks, and SCEs was also demonstrated in mammalian cells [143,144,145].

Safrole′s mutagenic potential was also studied in vivo [4]. In 1972, Epstein and colleagues obtained negative results for safrole in a mouse dominant lethal assay [146]. In line with this, testing of safrole in a bone marrow micronucleus assay and in a rat liver UDS assay also led to negative outcomes [147,148].

However, other studies clearly indicated the mutagenic potential of safrole in vivo. The first studies performed by Green and Savage in 1978 showed that safrole was positive in an in vivo i.p. host-mediated assay with *Salmonella typhimurium* [138]. Similar findings were published by Poirier and de Serres in 1979, utilizing the same assay with *S. typhimurium* or *Saccharomyces cerevisiae* [141]. Some years later, Daimon and colleagues showed that repeated-dose treatment of F344 rats with 125 or 250 mg safrole/kg bw dose-dependently induced chromosome aberrations in rat liver cells [127]. Moreover, singe-dose treatment of rats with 10–500 mg safrole/kg bw caused SCEs in rat livers in a dose-dependent manner, too. These effects were associated with the generation of DNA adducts in the hepatocytes of these rats [127].

The aforementioned indication of safrole′s mutagenic activity was substantiated by the findings of Jin et al., who observed an increased *gpt* mutant frequency in transgenic *gpt* delta rats after a 13-week exposure to safrole via diet at the highest dose group tested (0, 0.1%, 0.5%; *n* = 10/group, both sexes). The authors concluded that these data clearly demonstrated the mutagenicity of safrole in vivo [149]. These findings were confirmed by results of another study performed in 2013, utilizing a similar in vivo transgenic rodent model [150]. In this study, male F344/NSlc-Tg (*gpt* delta) rats (*n* = 15 per dose) were fed with 0.5% safrole via diet for 4 weeks. This dose was identified as a carcinogenic dose from an earlier study [140]. In this experimental setting, safrole caused a significantly increased *gpt* mutant frequency, which was associated with a tumor-promoting activity, as suggested by an elevated number and area of GST-P-positive foci in rat livers, compared to controls. The authors stated that these data confirm that safrole is a genotoxic carcinogen [150].

In contrast to safrole, data on myristicin′s mutagenic potential is sparse. A study published by Damhoeri et al. in 1985, studied the mutagenic activity of oleoresins prepared from myristicin-containing nutmeg fruits without metabolic activation in an in vitro mutagenicity assay in *S. typhimurium*. The authors reported that the tested oleoresins were mutagenic. Moreover, pure myristicin was also positive in the mutagenicity test [151]. Based on these data, it was suggested by Hallstrom and Thuvander in 1997 that both nutmeg and myristicin may be weakly mutagenic, but additional studies were required to finally conclude on the mutagenic potential [24].

Just recently in 2019, NTP characterized the mutagenic potential of myristicin. Myristicin was not mutagenic in *S. typhimurium* with or without metabolic activation. In addition, a micronucleus test was integrated in the subchronic toxicity study in which myristicin (0, 10, 30, 100, 300, or 600 mg/kg bw; 5 days/week) was administered via gavage to F344/NTac rats and B6C3F1/N mice (10 male and 10 female/group) for 13 weeks. There was a significant dose-dependent decrease in the percentage of polychromatic erythrocytes (PCEs) in the peripheral blood of male and female mice, illustrating toxicity to the bone marrow in mice and suggesting that the test compound reached the target tissue. In mice, however, no significant effect of myristicin on micronucleated red blood cells was observed. A significant increase in micronucleated immature erythrocytes in the peripheral blood was observed in male and female rats of the highest dose group (600 mg/kg bw). This was accompanied by significantly elevated amounts of circulating PCEs. Therefore, the authors suggested that myristicin might have stimulated erythropoiesis in rats. It was concluded that studies performed by the NTP provide limited evidence for the genotoxicity of myristicin [152]. However, findings from others indicated that myristicin, similar to other genotoxic alkenylbenzenes, e.g., safrole and methyleugenol, forms DNA adducts in vivo [100,130,132]. However, NTP authors stated that the consequence of these adducts is unknown, as myristicin was not tested for mutation induction in vivo [152]. Therefore, further and more adequate studies are needed to allow for a conclusive evaluation of the mutagenic potential of myristicin. Ideally, those studies should be designed as comparative studies (e.g., testing of myristicin vs. other alkenylbenzenes, such as safrole, in a similar experimental setting, e.g., as proposed by Nohmi and colleagues [150,153]), to allow a ranking regarding the genotoxic potential of these substances. As demonstrated with other alkenylbenzenes, in vivo assays capable of detecting gene mutations, i.e., transgenic rodent assays like the *gpt* delta assay, might be appropriate test systems for detecting potential alkenylbenzene-induced mutations.

#### 3.2.3. Genotoxic Effects in Pregnant Mice and in Offsprings

Since the altered hormone constitution in pregnancy may profoundly affect the activity of maternal xenobiotic metabolizing enzymes [154], a period of heightened susceptibility to chemical carcinogenesis may exist not only for the developing conceptus, but also for the dam [155,156]. For example, the effects of pregnancy on the covalent binding of several carcinogens to DNA were investigated in mice. Non-pregnant or timed-pregnant (18th day of gestation) mice of similar age were treated with safrole or 1′-hydroxy-safrole per os. Tissue DNA adduct levels at 24 h after treatment were analyzed via ^32^P-postlabeling. Binding of safrole and its proximate carcinogen, 1′-hydroxy-safrole, to maternal liver and kidney DNA was increased by a factor of 2.3–3.5 during pregnancy in mice [157]. In 1993, Randerath et al. observed a similar effect in the liver of pregnant mice exposed to myristicin (48,000 adducts/10^9^ nucleotides in liver DNA from dams vs. 17,000 adducts/10^9^ nucleotides in liver DNA from non-pregnant mice) [101]. This indicates that exposure to genotoxic compounds may be more hazardous for the maternal body during pregnancy than for non-pregnant adult females. In addition, safrole and myristicin may not quantitatively react in a first pass manner in mouse maternal liver alone. Part of the amount of safrole administered maternally and some reactive metabolites may reach the fetus transplacentally. Indeed, DNA adduct formation was observed by Randerat et al. in fetal liver after exposure to myristicin in pregnant mice [101]. The ability to form DNA adducts of myristicin transplacentally is of concern with respect to rapid cell divisions occurring in fetal liver cells, thus increasing the possibility of fixing potential mutagenic lesions which may further lead to carcinogenesis. In this context, administration of safrole to pregnant mice during the second half of gestation also led to the development of epithelial kidney tumors in female offsprings, demonstrating transplacental carcinogenesis [155]. In this study, a strong age- and sex-dependent difference (*p* < 0.01) in offspring renal carcinogenesis by safrole was observed. For comparison, in the case of the direct alkylating carcinogen ethylnitrosourea, no significant sex-dependent differences were observed [158], and preweaning as well as adult mice were equally sensitive to renal carcinogenesis by ethylnitrosourea [159].

### 3.3. Carcinogenicity of Safrole, Methyleugenol, Myristicin and Elemicin

Mutagenicity may lead to the development of cancer. For example, mutations in tumor suppressor genes or proto-oncogenes can cause uncontrolled cell division [160,161].

Safrole and methyleugenol are known hepatocarcinogens in experimental animals [126,162,163,164,165,166,167]. This was demonstrated by several rodent studies described and discussed in detail elsewhere [3,4,122,168].

In contrast to safrole and methyleugenol [92,122,168,169], data on the carcinogenicity of myristicin and elemicin are sparse. However, some limited experimental information is available suggesting the possible carcinogenic activity of these compounds [24,152,170].

Although results from an early experimental study using a preweaning mouse model suggest that myristicin is not hepatocarcinogenic [166], the reliability of this study must be questioned. Based on an in silico analysis, Auerbach et al., in 2010, reported that myristicin might potentially act as a weak carcinogen [170]. They predicted that administration of myristicin at 2 mmol/kg/day for 2 years would lead to a weak, albeit significant, increase in hepatic tumor burden in male rats. However, it should be noted that the informative value of in silico testing, with respect to the endpoint carcinogenicity, is rather limited [171].

For elemicin, first indications of tumorigenicity were reported by Wiseman and colleagues, who administered male B6C3F1 mice i.p 1′-hydroxy-elemicin or 1′-acetoxyelemicin in 4 doses during the first 21 days postnatally [126]. In this study, an average of 0.8 hepatoma/mouse relative to 0.1 hepatoma/mouse for the solvent-treated controls was observed after 13 months. An earlier and similar assay with 1′-hydroxy-elemicin, but using only 50% of the doses used by Wiseman et al., however, provided no evidence for its hepatocarcinogenicity when administered to preweaning male mice [166]. Data from two-year combined toxicity and carcinogenicity studies do not exist so far, neither for myristicin nor for elemicin. Those studies are crucial for a conclusive evaluation of the carcinogenic potential of myristicin and elemicin, as also stated by others [10,24,61]. Thus, the possible carcinogenic potential (including the underlying mode of action) of myristicin and elemicin merit further attention.

### 3.4. Other Toxicological Endpoints

In the following part of the manuscript, further toxicologically relevant effects of methyleugenol, elemicin, safrole, and myristicin will be described in a comparative manner. This includes acute, as well as subchronic toxicity, studied in vivo.

#### 3.4.1. Acute Toxicity of Methyleugenol vs. Elemicin and Safrole vs. Myristicin

In 2000, results of a short-term animal study done by NTP showed that methyleugenol is moderately toxic following a single oral dose. The median lethal oral dose (LD_50_) was 810 to 1560 mg/kg body weight (bw) for rats and 540 mg/kg bw for mice [92]. The undiluted chemical (98% purity) was found to be neither an eye irritant nor a skin irritant to rats and mice [92,172]. In contrast to methyleugenol, there is currently no literature available regarding the acute toxicity of elemicin.

Safrole was shown to be moderately toxic [173]. Its LD_50_ following oral administration was 1950 mg/kg bw and 2350 mg/kg bw in rats and mice, respectively [174,175]. Moreover, for safrole, acute neurological effects were described, including depression, ataxia in rats, as well as psychoactive and hallucinogenic effects in humans, which were considered as being similar to those reported for other methylendioxybenzene compounds, including myristicin [173,176,177]. The availability of literature regarding acute toxicity of myristicin is limited. In 1961, Truit and colleagues performed an acute toxicity study in rats treated i.p. with myristicin (200–1000 mg/kg bw) [178]. In the highest dose group, myristicin induced hyperexcitability followed by central nervous depression in rats. From these data, authors derived an LD_50_ > 1000 mg myristicin/kg in rats following i.p. application [178]. Although the database on myristicin is rather limited, its acute toxicity after oral administration was considered to be low [24]. Taken together, acute toxicity of myristicin seems to be comparable to that of safrole, especially regarding neurological effects.

#### 3.4.2. Subchronic Toxic Effects of Methyleugenol vs. Elemicin and Safrole vs. Myristicin

In 2000, NTP published the results of 14-week rat and mouse studies, in which subchronic toxicity of the oral administration of methyleugenol (0, 10, 30, 100, 300 or 1000 mg/kg bw via gavage; 5 days/week) to male and female F344/N rats and B6C3F1 mice was investigated [92]. Regarding the experiments done with rats, all animals survived until the end of the study. However, exposure to methyleugenol reduced body weight gain and caused cholestasis, hepatic dysfunction with hypoproteinemia and hypoalbuminemia, as well as atrophic gastritis. Moreover, this led to increased liver and testis weight and adrenal gland hypertrophy [92]. A no observed effect level (NOEL) of 30 mg/kg bw per day was identified [3,92]. In the mouse study, 9 out of 10 males and all females of the highest dose group died before the end of the study [92]. Methyleugenol exposure was associated with reduced body weight gain, elevated liver weight in mice, and increased incidences of cytological alteration, necrosis, bile duct hyperplasia, and subacute inflammation in livers. Furthermore, there were increased incidences for atrophy, necrosis, oedema, mitotic alteration, and cystic glands of the fundic region of the glandular stomach in mice of both sexes [92]. A NOEL of 10 mg methyleugenol/kg bw and day was identified for mice [3,92]. In sum, the available subchronic studies indicated that methyleugenol is moderately toxic, which includes different adverse effects, primarily in liver and stomach [3,92,179].

In contrast to methyleugenol, there is currently no literature available regarding elemicin′s subchronic toxicity.

In 1965, Hagan et al. performed a subchronic toxicity study, in which safrole (250, 500 and 750 mg/kg bw per day) was administered via gavage to Osborne–Mendel rats of both sexes for 105 days [180]. In the two highest dose groups, several rats died before the scheduled end of the study. In the lowest dose group, all rats survived until the end of the study. Several organotoxic effects were observed in this rat study, including liver hypertrophy, focal necrosis with slight fibrosis, steatosis, bile duct proliferation, and adrenal enlargement with fatty infiltration [4,180].

Comparable findings were obtained by Jin and colleagues in a rat study performed in 2011 [149]. In this study, safrole was administered to rats via diet (doses: 0, 0.1, and 0.5%; *n* = 10/group; both sexes) for 13 weeks. The main findings of this study were significantly reduced final body weights in male and female rats of all dose groups and hepatotoxic effects, including increased relative liver weights and significantly increased incidences of centrilobular hypertrophy, centrilobular vacuolar degeneration, and single cell necrosis of hepatocytes. Moreover, the authors found that the relative kidney weights of male and female rats were significantly increased after 13 weeks. Accompanying this, different nephrotoxic effects were observed in male rats of the highest dose group, such as significantly increased incidences of tubular hyaline droplets, granular cast, pelvic calcification, and interstitial cell infiltration in the kidney [149]. Taken together, the liver and kidney appeared to be the target organs with the most severe effects.

Regarding myristicin′s subchronic toxicity, NTP published in 2019 the results of 90-day toxicity studies performed in F344/NTac rats and B6C3F1/N mice [152]. In these studies, different doses of myristicin (0, 10, 30, 100, 300, or 600 mg/kg bw) were administered via gavage 5 days/week for 13 weeks to rats and mice of both sexes (*n* = 10). In the rat study, all males survived until the end, whereas, three female rats of the highest dose group died within 4 days of the study [152]. Exposure of rats to myristicin led to various treatment-related effects, including reduced mean body weight, enlarged livers, increased relative liver and kidney weights, as well as increased triglycerides and alanine aminotransferase activity regarding clinical pathology. Accompanying this, several treatment-related lesions were identified in rats, such as centrilobular hepatocyte hypertrophy and necrosis in the liver; epithelium atrophy and hyperplasia as well as necrosis in the glandular stomach; and renal tubule hyaline droplet accumulation as well as a slightly increased severity of nephropathy [152]. Moreover, myristicin also affected the reproductive system of male rats, which included decreased absolute left cauda and left epididymis weights, as well as a lowered number of sperm per cauda epididymis, germinal epithelium degeneration, elongated spermatid retention in seminiferous tubules of the testis, and exfoliated germ cells in epididymal duct lumina. Therefore, the authors concluded that oral myristicin exposure exhibited the potential to induce reproductive toxicity in male F344/NTac rats [152]. In the mouse study, all animals survived until the end. In mice exposed to myristicin, mean body weights were reduced, livers were enlarged, absolute and relative liver weights were elevated and hematology parameters were affected, which included increased leukocyte counts and segmented neutrophil number. Moreover, various treatment-related lesions were observed in mice, such as oval cell hyperplasia, centrilobular hepatocyte hypertrophy, and necrosis of the liver, epithelial and nerve atrophy, glands hyperplasia, hyaline droplet accumulation, and cytoplasmic vacuolization of the respiratory epithelium in the nose. Beside this, there was a significantly increased incidence of atrophy and hyperplasia in the epithelium of the glandular stomach as well as of chronic and epithelial suppurative inflammation in the forestomach [152]. From these findings, authors concluded that the major targets after oral myristicin administration in rats and mice were the liver and glandular stomach. Additional targets were salivary glands, the nose, kidney, testis, epididymis, and the forestomach. Study authors identified a lowest observed effect level (LOEL) of 30 mg/kg bw (increased relative liver weight) for male rats, 10 mg/kg bw (clinical chemistry) for female rats, 100 mg/kg bw (increased liver weights) for male mice, and 10 mg/kg bw (increased liver weights) for female mice. Moreover, a NOEL of 10 mg/kg bw for male rats and of 30 mg/kg bw for male mice was identified, but not for female rats or mice [152]. Together, the aforementioned data clearly indicate that the spectrum of toxic effects following subchronic myristicin exposure is at least in part, and especially regarding the hepatic and renal effects, comparable to that of the structurally similar compound safrole.

## 4. Conclusions

The limited toxicological data and the lack of occurrence and consumption data preclude a comprehensive evaluation of adverse health effects potentially associated with myristicin, elemicin, and other alkenylbenzenes.

Therefore, additional occurrence data is needed for all toxicologically relevant alkenylbenzenes in different food products, especially those containing high levels of alkenylbenzenes (e.g., essential oils, basil-containing pesto, or PFS) [5,11,58]. Alkenylbenzenes can be separated either via GC or high-performance liquid chromatography techniques (HPLC) followed by MS [12,25,181,182,183,184]. However, to increase the specificity and accuracy of methods used for sample preparation, extraction, as well as substance separation constant standardization efforts are needed. Furthermore, data on the consumption of alkenylbenzene-containing foods is required. This data should be collected via appropriate consumption surveys.

The alkenylbenzenes safrole and myristicin as well as methyleugenol and elemicin are structurally closely related (Figure 1). This in turn suggests that the hazard potential of those compounds could exhibit similarities. In this regard, it appears reasonable to identify potential hazards of the toxicologically widely unexplored alkenylbenzenes myristicin and elemicin in comparison to those of the known genotoxic carcinogens safrole and methyleugenol. The available toxicological data, e.g., data on toxicokinetics and genotoxicity, already suggest that both myristicin and elemicin might form reactive metabolites being similar to those being formed from safrole and methyleugenol. However, the sparse data also indicate that there might be quantitative differences that may result in an altered toxicity profile. This in turn, cannot be finally evaluated at present. Indeed, their genotoxic and carcinogenic potential is widely unknown, so far. In this context, two-year combined oral toxicity and carcinogenicity studies are mandatory for the evaluation of the long-term effects, as well as of the carcinogenic potential of myristicin and elemicin, as also recommended by others [10,24,61]. Moreover, the underlying modes of action of these compounds merit further attention, too. In this context, an appropriate experimental setting should be designed taking into account the alkenylbenzene-specific bioactivation (e.g., via SULTs) discussed in detail before [5].

It is important to note that the conventional bacterial reverse mutation test (Organization for Economic Co-Operation and Development (OECD) Test Guideline (TG) 471; Ames test [185]) lacks the metabolic competence to yield the ultimate carcinogenic sulf-oxy intermediates from alkenylbenzenes [186]. However, genetic modifications of the bacteria enabling SULT expression may lead to a more adequate in vitro setting for the mutagenicity testing of compounds metabolically activated via this pathway, such as methyleugenol, myristicin, and elemicin [5,186]. Substantiating this, Monien et al. demonstrated in 2011 that furfuryl alcohol was negative in the standard Ames test, whereas it was mutagenic in a modified setting utilizing *S. typhimurium* TA100 engineered for the expression of human SULT1 [187]. In line with this, in 2016, Honda and colleagues found methyleugenol, which is not mutagenic in standard Ames test [92], to be mutagenic in a modified Ames test using a human SULT1-expressing *S. typhimurium* TA100 strain [186]. Although scientific approaches exist that augment bacteria with human sulfotransferases, these systems are not yet internationally standardized and validated for regulatory purposes.

An alternative approach is the hypoxanthine guanine phosphoribosyltransferase (HPRT) assay (OECD TG 476), which is an in vitro mammalian cell gene mutation test using the *hprt* and *xprt* genes for gene mutation measurement in mammalian cells [188]. The method is described in detail elsewhere [189]. Modification of the HPRT assay via the use of replication competent cells (e.g., human liver cells) expressing human SULT1A1 could also offer an appropriate setting for in vitro mutagenicity testing of compounds bioactivated in a SULT-dependent manner, such as safrole, methyleugenol, myristicin, and elemicin.

From a toxicological point of view, and for the sake of animal welfare, initial mutagenicity testing of alkenylbenzenes with unknown modes of action, such as elemicin and myristicin, should be done in vitro. This might be sufficient, if initial testing of mutagenicity is conducted using appropriate test systems, enabling the intracellular activation to reactive sulfate esters by SULT-proficient bacterial or mammalian cells. For regulatory purposes, it appears however reasonable to recommend transgenic rodent (TGR) models (OECD TG 488 [190]) as ultimate confirmatory assays to decide on mutagenic potencies of alkenylbenzenes in vivo following a positive in vitro finding [189].

A promising candidate among those TGR models appears to be the *gpt* delta rodent gene mutation assay, developed by Nohmi et al. [153,191,192,193]. Since its development, it was already successfully used in various studies in the context of food safety research [153,193]. Regarding alkenylbenzenes, the *gpt* delta TGR model was demonstrated to reliably identify safrole, methyleugenol, and estragole as mutagens [124,149,194], as also concluded by others [3,4,195]. One additional benefit of such test systems is the option to evaluate mutagenicity in any tissue of interest [189]. This is of particular interest when mutagenicity would have to be tested in distinct organs, such as in the liver, e.g., for testing of suspected hepatocarcinogens, such as methyleugenol and elemicin [189]. Moreover, such an approach might pave the way for simultaneous testing of mutagenicity in different tissues at the same time. Moreover, such in vivo assays are needed to distinguish between genotoxic (e.g., aflatoxin B1) and non-genotoxic carcinogens (e.g., 3-chloro-1,2-propanediol) [153].

Together, the aforementioned approaches would shed more light on the existing, and currently still serious, data gaps, and could help to reduce considerable uncertainties currently impeding the evaluation of adverse health effects potentially associated with the consumption of foods containing alkenylbenzenes.

## Figures and Tables

**Table 2:** Human cytochrome P450 isoenzymes mediating the 1′-hydroxylation of alkenylbenzenes.

Substance	Cytochrome P450 Subtype	Reference
Methyleugenol	CYP1A2 (CYP2C9, 2C19)	[90,92]
Elemicin	CYP1A1, CYP1A2, CYP3A4	[88]
Safrole	CYP2A6 (CYP1A2, CYP2C19, CYP2E1)	[89,91]
Myristicin	CYP3A4 (CYP1A1)	[93]

Main human CYPs and, in brackets, contributing human CYPs involved in the metabolism of methyleugenol, elemicin, safrole, and myristicin.

## Data Availability

Not applicable.
